# Challenges and progress of neurodrug: bioactivities, production and delivery strategies of nerve growth factor protein

**DOI:** 10.1186/s13036-023-00392-2

**Published:** 2023-12-04

**Authors:** Nan Zhou, TingWei Gu, Yang Xu, Yuda Liu, LiHua Peng

**Affiliations:** 1 College of Pharmaceutical Sciences, Zhejiang University, 866# Yuhangtang Road, Hangzhou, 310058 PR China; 2 State Key Laboratory of Quality Research in Chinese Medicine, Macau University of Science and Technology, Macau, PR China; 3 Jinhua Institute of Zhejiang University, Jinhua, Zhejiang 321299 PR China

**Keywords:** Nerve growth factor, Pharmacological effects, Synthesis methods, Delivery strategies

## Abstract

Nerve growth factor (NGF) is a vital cytokine that plays a crucial role in the development and regeneration of the nervous system. It has been extensively studied for its potential therapeutic applications in various neural diseases. However, as a protein drug, limited natural source seriously hinders its translation and clinical applications. Conventional extraction of NGF from mouse submandibular glands has a very high cost and potentially induces immunogenicity; total synthesis and semi-synthesis methods are alternatives, but have difficulty in obtaining correct protein structure; gene engineering of plant cells is thought to be non-immunogenic, bioactive and economical. Meanwhile, large molecular weight, high polarity, and negative electrical charge make it difficult for NGF to cross the blood brain barrier to reach therapeutic targets. Current delivery strategies mainly depend on the adenovirus and cell biodelivery, but the safety and efficacy remain to be improved. New materials are widely investigated for the controllable, safe and precise delivery of NGF. This review illustrates physiological and therapeutic effects of NGF for various diseases. Moreover, new progress in production and delivery technologies for NGF are summarized. Bottlenecks encountered in the development of NGF as therapeutics are also discussed with the countermeasures proposed.

**Supplementary Information:**

The online version contains supplementary material available at 10.1186/s13036-023-00392-2.

## Introduction

**Fig. 1:**
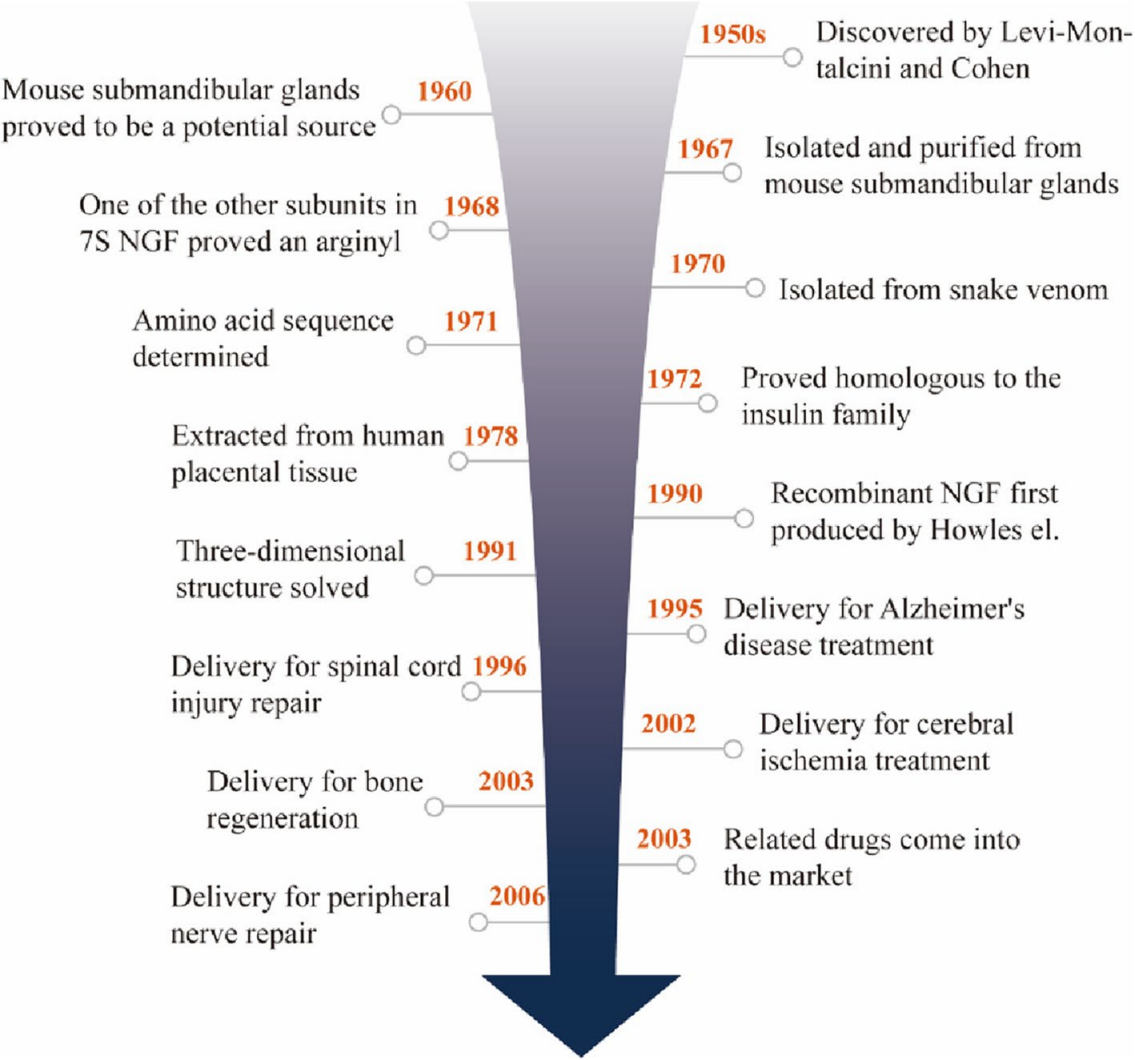
Historical development of NGF protein

**Fig. 2:**
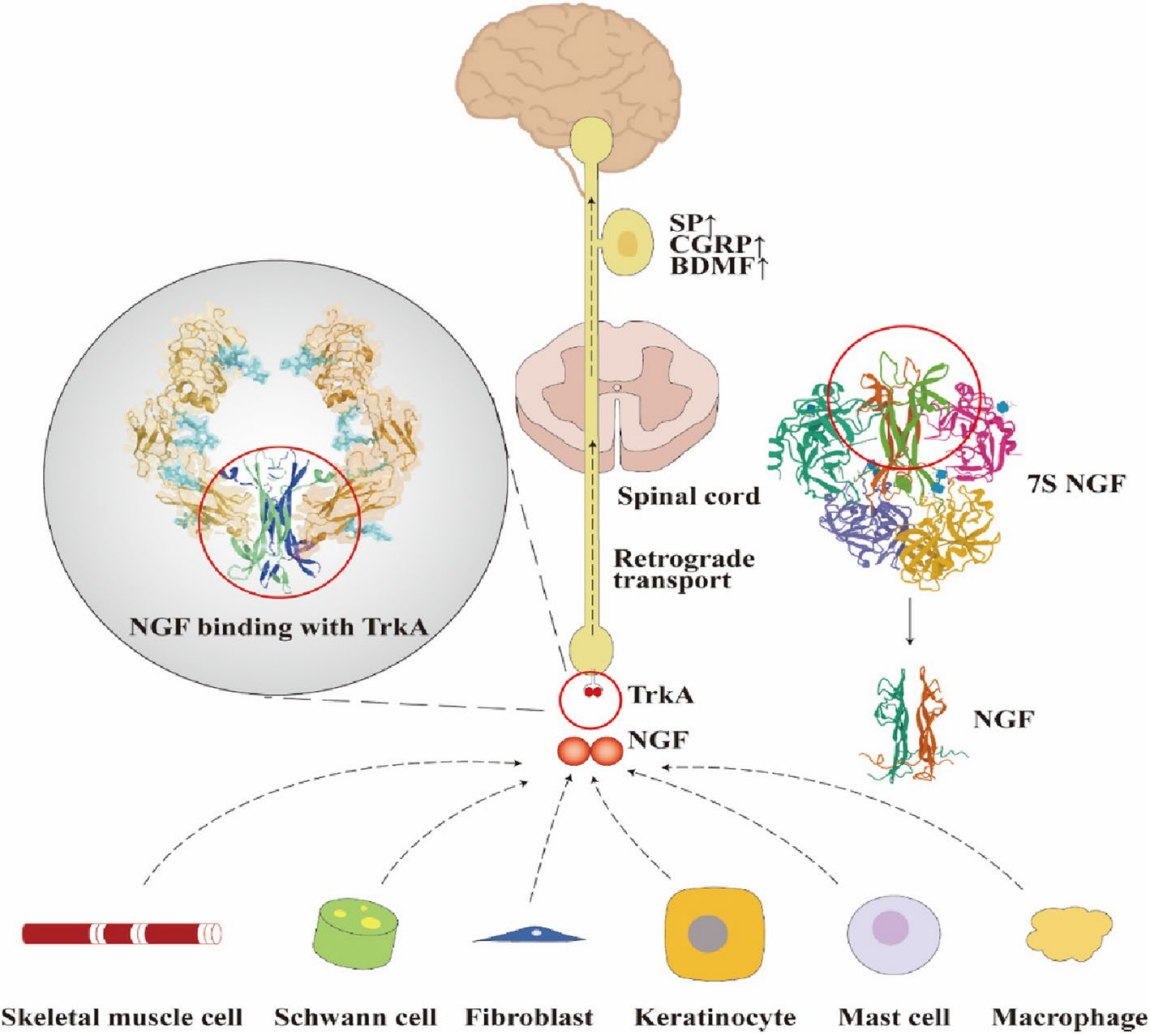
Structure, biosynthesis and retrograde transport of NGF

Since Levi-Montalcini and Cohen first discovered and purified nerve growth factor (NGF) in the 1950s and obtained NGF antibody in the 1960s, the research on the protein structure and physiological function of NGF has made rapid progress (Fig. 1). The structure of NGF protein was discovered to be homologous to insulin family and the complete three-dimensional structure was solved twenty years later [1]. As a member of the neurotrophic factor family, NGF can promote the growth, development and differentiation of central and peripheral neurons. It also helps maintain the normal function of the nervous system and accelerate the repair of the nervous system. It also promotes angiogenesis and functions outside of the nervous system. NGF is produced and utilized by several cell types, including structural, accessory, and immune cells through retrograde transported to the central nervous system by retrograded neuronal transport, regulating downstream cascade reactions by binding to p75 and TrkA receptors (Fig. 2). However, under pathological conditions, the secretion of NGF is frequently restricted, thereby affecting the normal functions of the nervous system. Exogenous NGF has shown excellent therapeutic efficacy in cell experiments in Alzheimer's disease, Parkinson's disease, hemorrhagic stroke, diabetic peripheral neuropathy, optic nerve injury and other disease models [1–6]. However, the clinical application of NGF is seriously hindered by the limited availability of natural resources of NGF. Currently, NGF is mainly produced by extraction from mouse submandibular glands, which has very high cost and potential immunogenicity. Total chemical synthesis and semi-synthesis are demonstrated to be challenging in achieving the correct folded structure of NGF. Compared with other preparation methods, genetic engineering is relatively simple and inexpensive, with the potential to solve the problem of peptide chain folding and modification through the perfect transcription and translation.

System of recipient cells. Current clinical NGF-based therapies are mainly by injection, adenovirus transfection and recombinant cells [3, 7, 8], but their safety and efficacy remain to be improved (Table 1) [9]. As a protein with a large molecular weight, high polarity, and negative electrical charge, the delivery of NGF confronts great challenges. Besides, as a neurotrophic drug, the targeting effect of NGF in brain in degenerative neurological diseases such as Alzheimer's disease and Parkinson's disease is significantly impeded by the presence of the blood brain barrier. Despite the obvious effects of exogenous neurotrophic drugs have obvious effects in cell experiments in vitro, poor results in most animal and clinical trials, which are mainly contributed to the low delivery efficiency of NGF [10].

**Table 1:** Clinical trials of NGF for the treatment of AD

Type of study	Administration route and dose	Main outcomes
2020; Phase II (NCT00087789)	2 × 10^11^ vector genomes of AAV2-NGF by stereotactic inj	NGF did not directly reach cholinergic neurons due to limited spread and inaccurate stereotactic targeting
2018; Phase II (NCT00876863)	2.0 × 10^11^ vector genomes of AAV2-NGF delivered by bilateral stereotactic injections	No significant difference between the treatment group and placebo
2016; Phase I (NCT01163825)	Cell biodelivery expressing NGF 10 ng /device/day, targeting at the cholinergic basal forebrain	13 of 16 implants released NGF, 8 implants released NGF at the same rate or higher than before the implant procedure
2015; Phase II (NCT00017940)	Autologous fibroblasts based NGF gene therapy to the basal forebrain, 1.2 × 10^10^, 5.8 × 10^10^ or 1.2 × 10^11^ vector particles	In the degenerating neurons in the brain, trophic response to NGF in the form of axonal sprouting toward the NGF source
2014; Phase I (NCT00087789)	1.2 × 10^10^, 5.8 × 10^10^, 1.2 × 10^11^ vector genomes of AAV2-NGF	Long-term, targeted, gene-mediated NGF expression and bioactivity
2012; Phase I (NCT01163825)	Cell biodelivery expressing NGF 300 ng/10^6^ cells/24 h, targeting the NBM or the limb	Persistent NGF secretion was detected in half of the patients
2005; Phase I	Implanting NGF-expressing autologous fibroblasts into the forebrain, 2.5 × 10^6^ total cells to 10.0 × 10^6^ total cells	Improvement in the rate of cognitive decline

In this review, the physiological functions of NGF and its clinical applications in different disease areas are described in detail. In addition, the frontier progress of NGF drug delivery strategies and the biosynthetic system of recombinant NGF are highlighted, opening up possibilities for NGF biosynthesis, drug delivery and therapeutic applications for different diseases.

## Physiological and therapeutic effects of NGF

**Fig. 3:**
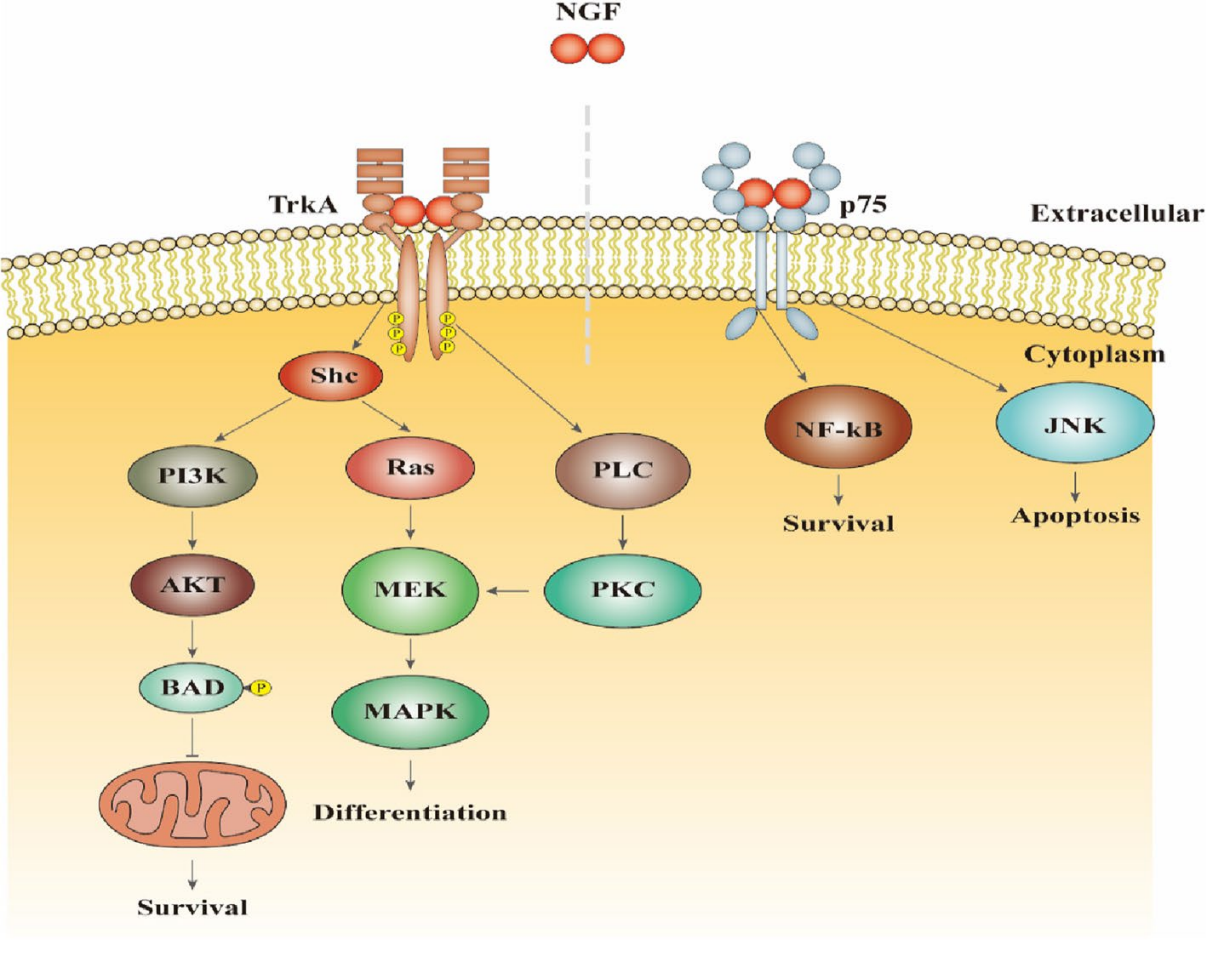
Receptors and physiological mechanisms of NGF

NGF performs physiological functions by binding to two types of receptors (Fig. 3). TrkA is the primary receptor for NGF, responsible for mediating its biological activity. It exhibits a slow binding rate to NGF to exert biological activity, slow in binding to NGF, but has a high affinity for the ligand. The other type of receptor is fast in terms of binding kinetics but has a low affinity with NGF, called p75 for short, which promotes the binding of TrkA to low concentration NGF thus regulates signaling through TrkA [11]. The binding of receptor and NGF initiates signaling cascades, dynamically regulating neuronal and non-neuronal behavior under physiological and pathological.

Conditions (Fig. 3). The combination of NGF and TrkA triggers activation of phosphatidylinositol 3-kinase (PI3K), mitogen-activated protein kinase (MAPK) and phospholipase C-γ (PLC-γ) signalling pathways involved in cell survival and differentiation. While binding to p75 not only mediates signaling through TrkA, but also regulates cell survival and apoptosis [11, 12]. During the injury repair period of neurons, NGF could promote the directional growth of nerve fibers, induce the development of axons and dendrites, and promote the mitosis, differentiation and repair of neurons. By promoting the growth of Schwann cells (SCs) and glial cells, NGF could repair the myelin sheath, protect damaged neurons from further damage, reduce the death of nerve cells, and support the survival of neurons [13, 14]. Moreover, NGF plays a significant role in the peripheral nervous system, regulating the metabolism of peripheral organs such as white adipose tissues [15]. 

### NGF for neurodegenerative disorders treatment

**Fig. 4:**
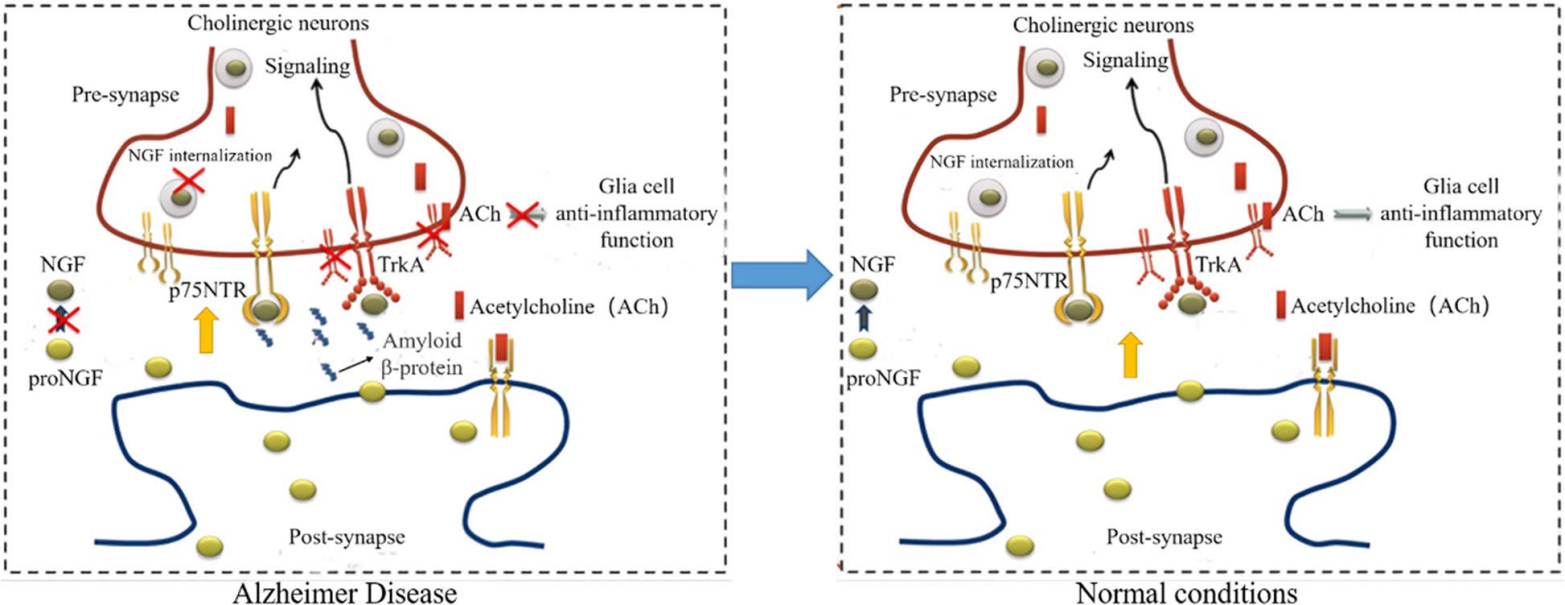
The treatment of NGF for Alzheimer’s disease. This figure was adapted from Figure 1 from Mitra et al. (2019) [77]

NGF plays an indispensable role in regulating nervous functions and have been explored for neurodegenerative disorders treatment for a long time. In some pathological conditions of the central nervous system (CNS), the endogenous level of NGF is decreased and their neuroprotective and neurotrophic properties are impaired [16, 17]. The use of exogenous NGF has been proven to complement the pathological absence of NGF and play the role in promoting the repair of nerve damage in vitro, making NGF a promising therapeutic agent for neural repair in the treatment of CNS diseases such as Alzheimer's disease (SCI) [18]. epilepsy [7],(AD) [4, (Fig. 4), epilepsy [4], and spinal cord injury (SCI) [19]. As for AD treatment, NGF has shown the capacity to improve neuronal damage, axon guidance, cell morphology, learning and memory deficits by inhibiting the hyperphosphorylation of tau protein in AD mice [2]. Since NGF does not cross the blood brain barrier, NGF administration through intracerebroventricular (ICV) route has been carried out to evaluate the safety and efficacy of NGF in Alzheimer's disease patients [2]. In the treatment of epilepsy, NGF can significantly reduce seizure onset, shorten seizure duration and alleviate neuronal loss in the epileptic brain [4]. The underlying mechanism is attributed to the activation of TrkA and inhibition of p75 receptor/Caspase-involved pathways. SCI is another inconvenient issue resulting in nerve disorders. NGF has been shown to promote the proliferation of spinal cord neural precursors/stem cells in vitro and in vivo, thereby being helpful in the treatment of SCI [4]. Besides, the neuroprotective effect of NGF raises the possibility of developing neuroprotective agents for cerebral ischemia, which is a novel approach to its treatment [4].

**Fig. 5:**
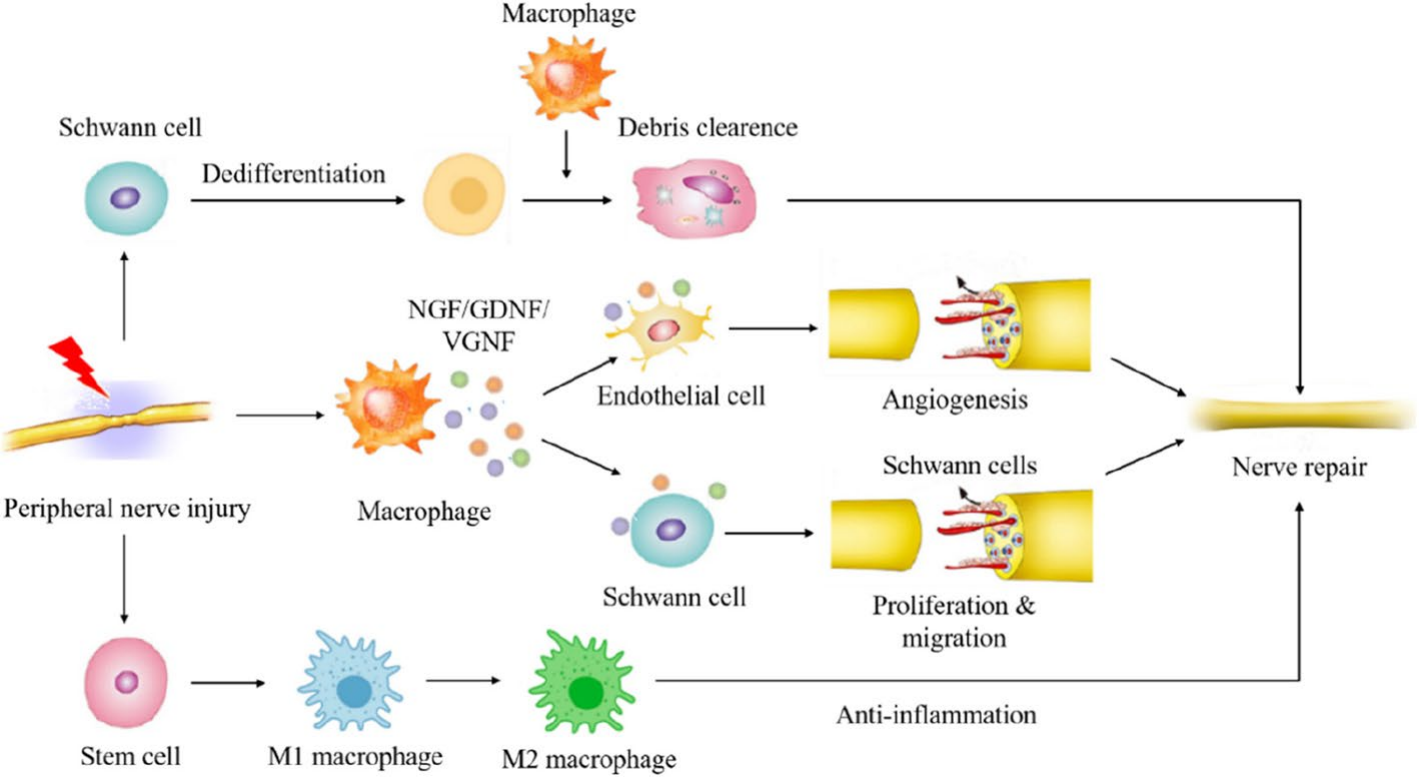
The treatment of NGF for peripheral nerve injury

The repair and reconstruction of peripheral nerve injury (PNI) has always been one of the clinical difficulties, especially for peripheral nerve defects larger than 3 cm [20]. Autologous nerve transplantation is considered to be the first choice for treatment, but it has many problems in clinical treatment, such as a lack of donors and donor site morbidity [20]. NGF has been proven to promote sciatic regeneration, which is a kind of PNI, in diverse rat models. According to the research of Li et al., NGF can promote the repair of injured peripheral nerves and accelerate the regeneration of axons and myelin sheaths by inhibiting endoplasmic reticulum stress, accelerating the clearance of myelin debris by SCs and reducing cell apoptosis in nerve tissue [18] (Fig. 5).

### NGF for optic nerve injury

**Fig. 6:**
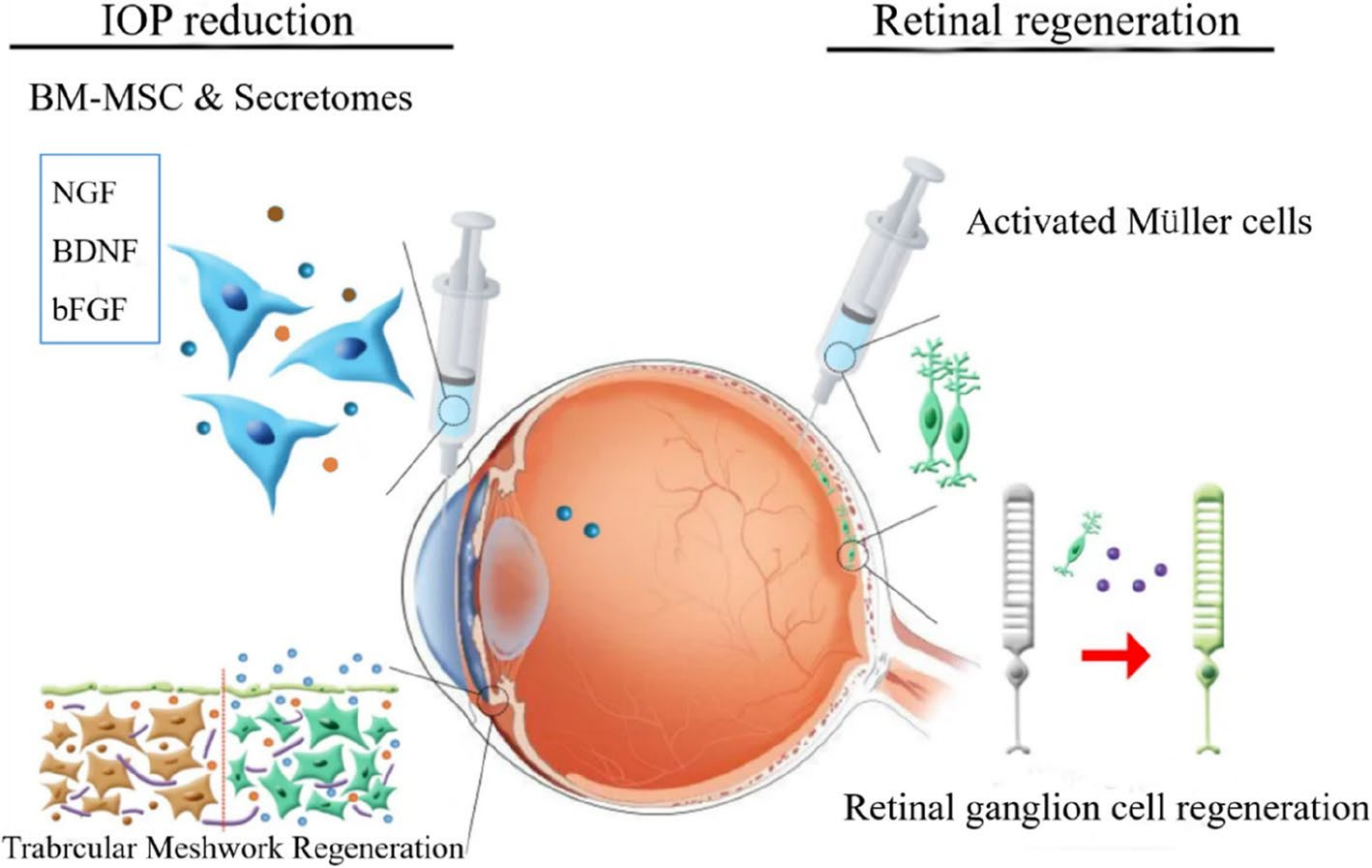
The treatment of NGF for optic nerve regeneration. This figure was adapted from Figure 4 in Kwon et al. (2020) [78]

The optic nerve originates from the ganglion cell layer of the retina and is composed of axons of retinal ganglion cells (RGCs). Like most neurons in the CNS, RGCs cannot regenerate themselves, so optic nerve damage caused by head trauma, ischemia, or glaucoma usually leads to permanent vision loss [6]. NGF has a certain effect on optic nerve regeneration (Fig. 6). p75, the receptor for NGF and other neurotrophins, plays a great role in the thalamocortical innervation to the visual cortex [6]. NGF can induce the modification of presynaptic elements in the adult visual system, prevent changes in the ocular dominance distribution of neurons in the visual cortical neurons and promote the functional recovery of RGCs after ischemia [6]. Currently, purified mouse nerve growth factor (mNGF) is often prepared into eye drops for local treatment of ophthalmic diseases [21–23]. Clinical case study reports show that mNGF could promote rapid healing of ulcers and had fewer systemic side effects in the treatment of corneal neurotrophic keratitis [24]. In addition, in the treatment of advanced glaucoma, mNGF could gradually ameliorate the function of the inner retinal layer and the nerve conduction in the posterior retina and the visual acuity of the patients was improved during the treatment even 3 months after discontinuation of treatment [24].

### NGF for skin wound healing

Increasing evidence shows that NGF can significantly promote wound healing. After topical application of NGF to the leg ulcers of patients with rheumatoid arthritis, the area of ulcers was observed to decrease rapidly [25]. At the same time, pain and inflammation were improved during treatment. The efficacy of NGF may be attributed to its promotion of keratinocyte proliferation and angiogenesis [25]. In addition, clinical studies on pressure ulcers found that, after the topical NGF treatment, the area of pressure ulcers on the elbow was reduced to the control group, and the rate of recovery is irrelevant to the severity of the ulcer, the patient’s age or the surgical site [16]. These results showed that the topical application of NGF in patients can promote the healing of ulcerated skin. For skin nerve repair, electrical stimulation assistance and smart wearable devices that release NGF can be considered [26, 27].

## Strategies for recombinant NGF production

**Fig. 7:**
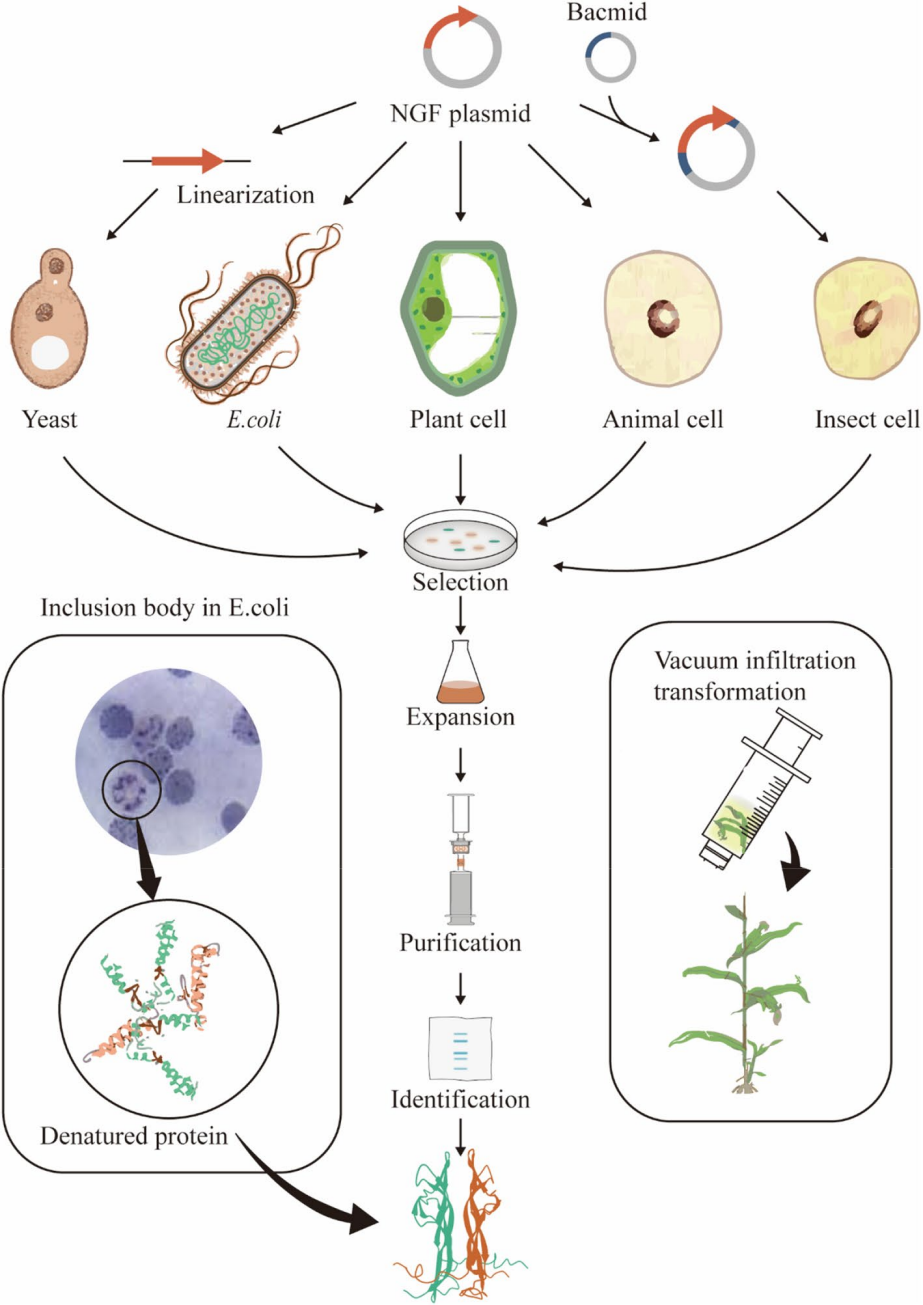
Synthetic biological methods used to synthesize hNGF in different host cells

As early as the 1950s, nerve growth factors were extracted from snake venom and mouse submandibular glands, respectively. The current commercial NGF still uses the original extraction method to produce mNGF. However, these agents are heterogeneous mixtures of partially degraded dimers and are not suitable for therapeutic purposes [28]. Furthermore, the inadequate supply of raw materials from mouse submandibular glands and complicated extraction technology, the yield of NGF falls far short of the clinical demand, leading to the rapid rise in the price of NGF. In addition, potential pathogens in mice also present various risks for NGF derived from the submandibular gland, and there is a concern about cross-infection with humans. The production of recombinant protein is the primary focus of protein production. Bioactive mature, fully processed NGF consists of a dimer of 13-kDa polypeptide chains, each with three intra-chain disulfide Bridges, highly similar to other proteins in the insulin family, which includes brain-derived neurotrophic factors (BDNF), neurotrophin-3 (NT-3), and neurotrophin-4 (NT-4) [29]. Many researchers have used synthetic biology methods to produce recombinant nerve growth factor in various expression systems, commonly referred to as bioreactors, to produce high purity, properly folded NGF without risk of pathogens (Table S1). Most researchers chose to yield human NGF, avoiding the potential immune risks that mouse NGF would have. Here we present previous attempts to recombine NGF production (Fig. 7), compare and contrast the features of different expression systems and future production directions.

### Protein engineering in *Escherichia coli*

In 1992, a concentration of 85 mg/L human nerve growth factor (hNGF) was obtained by Dicou [30], which is relatively high in the expression system of recombinant NGF. In that year, Dicou [30] and Kilmon [31] respectively expressed NGF in *E*. coli.

Kiyoshi et al*.* verified that hNGF aggregates in cells in the form of inclusion bodies. At this time, the expression strategy of *E*. *coli* was to extract inclusion body first and then refold the denatured proteins rather than secretory protein expression, and the difference in hNGF yield was dependent on the formation of inclusion bodies. They obtained 85 mg and 10–12 mg of recombinant human nerve growth factor (rhNGF) per liter of bacterial suspension before purification, respectively. However, 87% of the hNGF protein was lost during the purification process, which may indicate that the folding of hNGF folding is not entirely correct [30]. In 2001, Anke et al*.* [28] continued the method of extracting proteins from inclusion body, but changed the host strain to DE3, which is more commonly used by later researchers. The important role of pro-sequence in facilitating the folding of NGF in inclusion body is also demonstrated. Anke et al*.* verified that pro-sequence mediates protein entry into the endoplasmic reticulum and significantly increases the yield and rate of disulfide bond formation. Although protein production through inclusion body can ensure high protein yield, it is difficult to extract and purify protein in the later stage, involving disulfide bond formation and protein refolding [30, 31]. The inclusion bodies located in the cytoplasm are particularly susceptible to degradation. An improved strategy was to produce NGF in the periplasmic space, where the protease content of protease is lower and disulfide bond forming enzymes can catalyze correct disulfide bond formation [32, 33]. Different signal peptides are used for subcellular localization and increase in expression of recombinant proteins. Kilmon tried different N-terminal sequence of trp L and β-lactamase signal peptide, and it was found that the former achieves better expression of NGF [31]. Tilko used DsbA signal sequence, which is a disulfide bond forming enzyme, to express recombinant NGF [34], leading to soluble periplasmic expression of DsbA-NGF in *E*. coli, but yields using this approach are less than expression in cytoplasm [35].

In terms of protein expression level, NGF expression in *E*. *coli* expression system was the second highest (85 mg/L), only lower than that in the mammalian expression system (25 μg/L). Considering the low culture cost of *E*. *coli*, it has a bright prospect in the industrial production of recombinant proteins. The optimized medium parameters were determined including redox potential, L-Arginine concentration, pH value and IPTG concentration [28, 34]. Compared to medium parameters, whether the expressed protein are in the form of inclusion body or secreted protein contributes more to the yield and production. Inclusion body insures higher expression and yield but does not guarantee correct folding. Moreover, protein loss during purification results in low final production [30]. After nearly three decades of development, the production strategy of *E*. *coli* expression system developed from inclusion body extraction to secreted protein expression. The production of NGF as secreted protein in *E*. *coli* expression system needs to be further studied.

In most cases constructed plasmids were used to express the coding sequence of human NGF precursor as the introns in pro-sequence promotes disulfide bond formation and correct protein folding [28]. On the contrary, cDNA of mature NGF doesn’t result in correct post-translational modification, though it ensures a valid amino acid sequence. NGF expressed by precursor sequence exhibits biological activity, but the three-dimensional structure remained to be validated. *E*. *coli* expression system still confronts great challenge to express NGF with correct folding and structure as some eukaryotic protein-modified enzymes may be missing in *E*. *coli*, and there are differences in signaling pathways that regulate posttranslational modifications.

### Protein engineering in yeast

Akira et al. [36]*.* in 1992 designed an advanced δ-integration system and used it for NGF expression from Saccharomyces cerevisiae. MFα1 prepro-signal was constructed in plasmid to express protein allowing for secretion. MFα is processed in two steps: the signal sequence is cleft in the endoplasmic reticulum, and the resulting pro-α-factor is transported to the Golgi apparatus, where the leader sequence is removed. Haploid yeast strains were constructed with approximately 20 copies of an δ-integrated NGF expression cassette on four chromosomes. The strain secreted NGF at levels 3–4 folds (3-4 mg/L) higher than a 2 μm-based plasmid. The leader sequence played an important role in proper intracellular transport of NGF. Based on δ-integration system of Akira et al., other leading sequences were tested such as yeast invertase, and the results showed that MFα1 is most effective in promoting NGF secretion [21].

Since the 20th century, generational advances have been made in genetic components of yeast expression system. Modifications on the endosome-to-Golgi trafficking were found to effectively reduce protein retention besides increasing protein secretion and a fungal α-amylase was produced at a high yield of 2.5 g/L [37]. *Pichia pastoris*, a methylotrophic yeast, is another established system for the production of heterologous proteins. In 2009, the 9.43 Mbp genomic sequence of the GS115 strain of *P. pastoris* was presented and annotated. In 2006, the utility of *P. pastoris* was expanded by genetic engineering to secrete human glycoproteins by replacing four genes to heterologous genes allowing sequential steps of human glycosylation [38].

Since the twentieth century, no studies tried NGF production in yeast, although Pichia pastoris is the most commonly used engineering strain. It is not confirmed whether yeast is not suitable for NGF production. Yeast has most eukaryotic post-translational modifications and suitable for secreted protein expression, but some transcriptional levels or other post-translational modification processes can be further introduced by human proteases to promote correct protein translation and folding. For example, the high specificity of *P. pastoris* mannosylation usually leads to rapid clearance of recombinant drugs from the blood due to a lack of sialic acid glycosylation.

### Protein engineering in insect cells

NGF was cloned and expressed in baculovirus-infected Spodoptera frugiperda (SF-9) insect cell system as early as 1991 by Stephen et al. [39]. The culture supernatant contained 2–3 mg/L recombinant human NGF. After purification by affinity chromatography, they obtained 1–2 mg of pure, human NGF per liter of culture supernatant with a high recovery rate of about 60%, which is 2 to 3 times of that in *E*. *coli* system, though the expression level is lower than in E. coli.

Jim et al*.* also obtained NGF at a slightly higher concentration of 5-10 mg/L at a similar recovery in the same year [39]. Remarkably, at the protein verification level, they not only did NGF bioactivity analysis, isoelectric point analysis, amino acid sequencing, as most cases did, specific structural analyses such as dimer / monomer equilibrium analysis and carbohydrate analysis were also carried out. It's found that rhNGF molecules were homodimers and mature rhNGF was found not to be significantly glycosylated (< 0.08 mol of N-acetylglucosamine/mol of protein), so the advantage of insect expression system in glycosylation was not demonstrated. In addition, mass spectroscopy gave a molecular mass (13 258 Da) similar to that predicted for the mature monomer (13 261 Da), accounting for the presence of cysteine bonds. Isoelectric focusing gave a single band which ran at a pI of approximately 9.3, although the predicted pIs for recombinant human NGF is 8.8 while they did not attempt new genetic modification or fermentation strategies, they provided new structural information about the recombinant NGF expressed by insects. Mass spectroscopy gave a molecular mass (13 258 Da) similar to that predicted for the mature monomer (13 261 Da), accounting for the presence of cysteine bonds. Isoelectric focussing gave a single band that ran at a pI of approximately 9.3, although the predicted pIs for recombinant human NGF is 8.8 [39]. After that, Shelley et al. and Li Jianan et al. transfected the same cell line with virus as vector in 2001 and 2013 [17, 40]. Robertson et al*.* tried clinical grade recombinant NGF formulation for ADs, and the recombinant protein is stable in the formulation for at least three months at 78C, but there is considerable non-specific binding to the pump system [41].

In the insect cell expression system, the maximum NGF yield is 1–2 mg per liter culture broth, which is 3 to 6 times as much as *E*. *coli*, though the maximum concentration is lower than that of the *E*. *coli* expression system. Its expression cost is also much higher than that of *E*. *coli* and yeast, which does not reflect its advantages in the economy of production. Moreover, there was no data showing that the biological activity of the recombinant NGF was better than that of other expression systems, though experiments with insect expression systems provided more information on the structure of recombinant NGF. The advantage of insect expression system in glycosylation was not demonstrated, and the transfection process requires a large number of viruses. In addition, there is a disconnection between upstream construction and downstream separation and purification, more attention is paid to the efficient expression of upstream construction, but lack of consideration on whether the product can be effectively extracted and purified. So at the moment, insect expression systems don't offer a great advantage.

### Protein engineering in mammalian cells

The attempts to express recombinant NGF in animal cells started late but are extensive. Since 2000, a variety of animal expression systems have been attempted, ranging from cell lines to animal organs such as mammary glands and salivary glands. The highest yield of NGF was expressed in 2008 in rabbit mammary gland [42], which was 173.1 mg/L. They produced human NGF in rabbit milk by employing a recombinant adenoviral expression system. Using salivary glands as a bioreactor, Zeng Fang et al*.* obtained a total of 18 transgenic mice in 2017 [43]. Transgenic mice that secreted high levels of hNGF (1.36 mg/L) in saliva were selected. Approximately 28 μg of hNGF was purified from about 40 mL of saliva, resulting in a yield of 51.47%.

Several attempts have been made to express recombinant NGF blasts in stem cells and fibroblasts. In 2000, mammalian cells were first used to express NGF. A tetracycline-regulatable gene expression system was generated, and NGF-induced neurite outgrowth could be precisely controlled within 24 h [44]. In 2006, A multigenic Lentiviral vector was constructed to infect mouse NSC and stably express recombinant human NGF [22]. In 2008, rat MSCs were constructed to express NGF using an adenoviral vector [45]. In 2010, the recombinant expression of human NGF gene in rabbit MSCs was undertaken, and the cells were transfected using ProFection Mammalian Transfection system-calcium Phosphate [46]. Though the yield and concentration were limited, the value of stem cells expressing NGF lies in their potential cell delivery applications. Delivery of gene-modified stem cells to the site of injury may promote neural repair or regeneration and return of function after peripheral nerve or spinal cord injury, which requires specific formulation and cell delivery materials, such as scaffolds [25, 45, 47].

In HeLa Tet-off cells, NGF concentration in the medium reached 20.3 mg/L after two weeks of high-density culture, which is considered high for recombinant NGF genetic engineering in a laboratory scale [48]. Their rhNGF was shown to possess in vivo and pharmacological effects that are comparable with the mNGF, with no apparent side effects, such as allodynia. In CHO cells, Li Xu and Ana et al*.* attempted NGF production in 2014 and 2019 respectively [23, 49]. Among them, the concentration of NGF in the culture medium of Ana et al. reached the highest at 8.4 mg/L, and the concentration after purification was 0.031 g/L.

The greatest advantage of the mammalian cell expression system is its ability to efficiently amplify and expression of recombinant genes, and the highest concentration of human NGF can be expressed at 173.1 mg/L, which is the highest among all the expression systems. Cell lines such as HEK293 and CHO have been developed as efficient transient and stable expression systems, respectively, transfected with liposome, calcium phosphate or PEG as transfection reagents. But scaling up is technically and economically challenging. Currently, mammalian cells are the most attempted expression system for the expression of recombinant NGF. Theoretically, mammalian expression systems have the most similar protein translation process and are most likely to express correctly folded human NGF and have higher activity for human receptors. However, when it comes to promoting dorsal root ganglion and PC12 differentiation, there was no clear advantage in the activity of NGF expressed in mammalian cells. Mammalian expression systems are currently expensive to cultivate and may not be industrially friendly for the production of NGF unless further studies demonstrate significant laboratory and clinical advantages for mammalian expressed NGF.

### Protein engineering in plants

The production of recombinant proteins in plants is a new field [50]. The production of NGF in plants has not been reported, but a few patents have been published. There are four major plant-based expression systems available for the production of foreign proteins: Transgenic plants, chloroplast transformed plants, transient expression and plant cell suspension cultures [51]. Galban et al*.* in 2011 used Agrobacterium-mediated transformation to achieve not only transient transformation but also stable transformation in *Nicotiana benthamiana* to produce NGF [52]. Stability transformation avoids the need for costly bioreactor growth and maintenance systems. This makes its production system cost-effective and makes industrial scaling possible. Leaf discs are transformed and calluses formation is induced to obtain stable transformation cells. The obtained plants were self-crossed to the third generation to obtain stably transformed plants. As for plasmid construction, cDNA encoding pre-pro-NGF and leader sequence were inserted into the plasmid. The plasmid vector also contains sequences that regulate the entry of proteins into the endoplasmic reticulum. The yield of recombinant protein was 3 ~ 5 μg/kg both in stable transformation and transient transformation.

Wang Yueju et al*.* produced 0.28 mg/kg NGF in lettuce in 2019, much higher than the 0.03 mg/kg they obtained in tobacco using the same method [53]. Moreover, its protein activity was higher than 500 Au/ug, which exceeded the 150 Au/ug in the tobacco system. They demonstrated that the lettuce was an efficient NGF production system and the production cycle of lettuce was as short as 4 ~ 6 weeks, which was conducive to rapid production. In addition, they improved the Agrobacterium vacuum permeation method, which reduced leaf tissue necrosis compared to the method requiring longer vacuum exposure times.

The plant system offers practical, biochemical, economic and safety advantages compared with conventional production systems. Plants are already being used to produce antibodies, vaccines, growth factors and many other proteins of pharmaceutical importance [51, 54]. Molecular farming and bioreactor in plants has great potential, offering practical, biochemical, economic and safety advantages compared with conventional production systems [55, 56]. But at the present stage, it still needs to study how to improve the yield and expression efficiency, as well as purification technology.

## NGF delivery strategies for different diseases

**Fig. 8:**
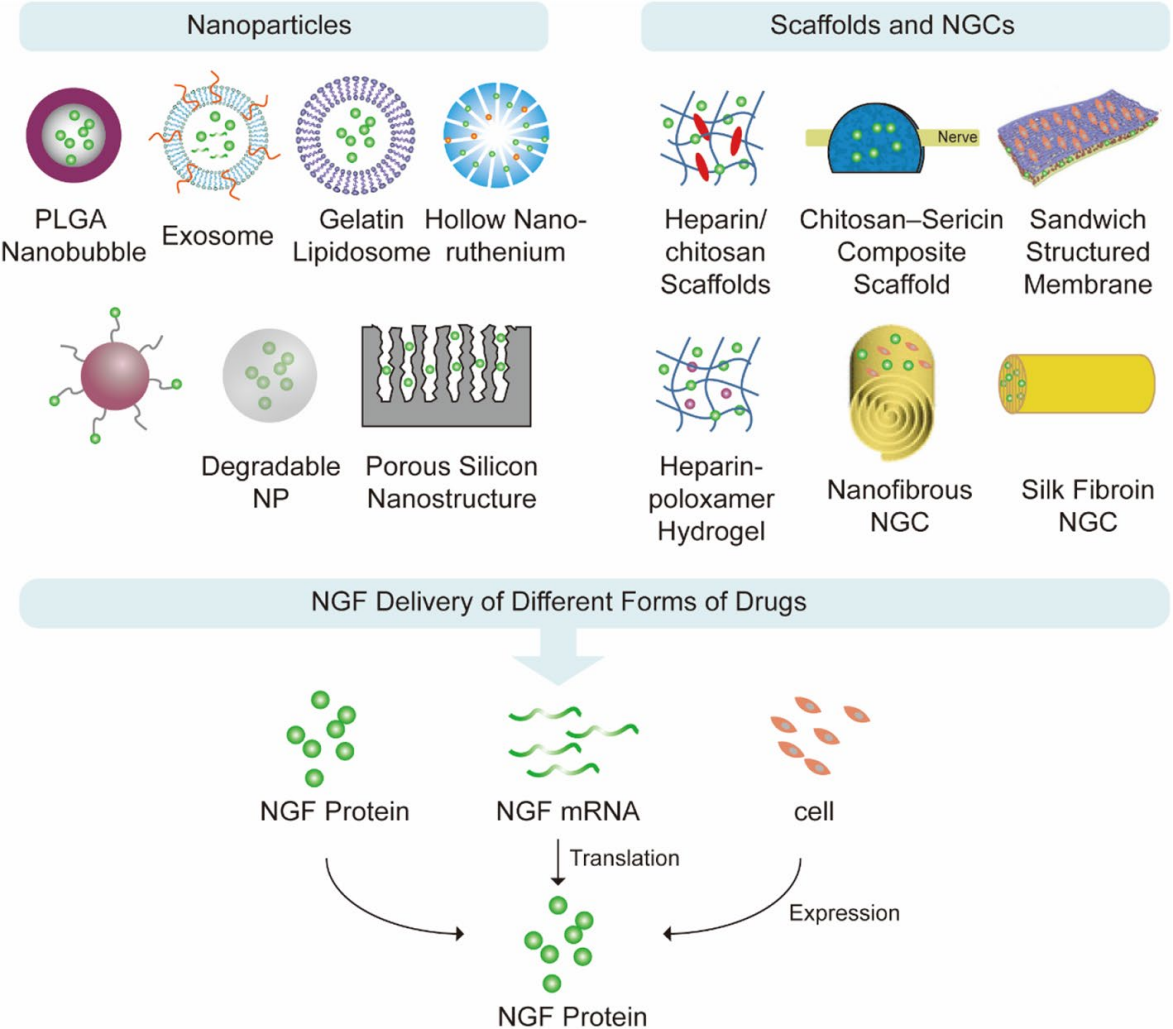
NGF based drug delivery vector and delivery strategy

NGF administration generally faces the problem of insufficient bioavailability upon systemic or topical delivery. Direct administration of NGF is limited by their rapid degradation and dilution around the injured sites. Especially, when utilizing naked NGF for brain nerve injury repair, the short half-life for circulation and the inability of NGF to cross BBB are the main obstacles to enriching NGF in the brain and achieving an optimal therapeutic effect. In practice, even moderate clinical effects require large and repeated doses, which is clinically impractical and expensive, and the low clinical trial pass rate for NGF is closely associated with the formulation and delivery methods (Table 1). Besides, NGF has biosafety risk. So, delivery strategies to enhance the bioavailability of NGF and reduce the administration dose and frequency to eliminate the potential cytotoxicity are greatly needed to be proposed. Therefore, new materials have been designed for NGF delivery, such as nanomaterials, bioactive scaffolds and neural conduits (Table S2), to achieve controlled release, sustained release, BBB breakthrough or targeted delivery (Fig. 8).

### Nanoparticles

In NGF drug delivery research, nanoparticles are the most widely used delivery vehicles. Multifunctional nanoparticles can be engineered using existing chemistries to obtain NPs with different sizes, targeting, and surface properties to deliver NGF in the form of protein or mRNA [57–60]. Brain targeting is challenging, and the BBB is the main problem that prevents this important functional protein from exerting its pharmaceutical effect. Naked NGF protein cannot pass BBB and has a short half-life, making it difficult to accumulate in the brain [61]. Brain-targeted delivery of NGF aims to achieve controlled and sustained release, improve the BBB breakthrough and treat degenerative neurological diseases such as AD. Intraventricular injection delivery based on nanoparticles/viral vectors and implantation of protein-generating cells all have certain effects on brain targeting [1, 62–65].

Considering the ability to cross the BBB, nanoparticles carrying NGF show advantages for treating brain nerve injury. NGF and iron oxide particles were co-encapsulated into crosslinked albumin nanocarries which were modified with apolipoprotein E on the surface. The nanocarriers could pass through an artificial BBB and release NGF, which induced neurite outgrowth in PC12 cells in vitro. Zhou et al*.* successfully prepared a novel flower-like hollow ruthenium nanoparticles loaded with NGF, and thermoresponsive phase change material was used as a switch to control the release of NGF [2]. Under near-infrared irradiation, the nanocomposites could pass through BBB due to the photothermal effect and enrich in the brain. They could inhibit TAU hyperphosphorylation, reduce oxidative stress, restore nerve damage and maintain neuronal morphology, thereby significantly improve learning and memory in AD mice. For the treatment of cerebral ischemia, Yang et al*.* engineered the exosomes to simultaneously deliver NGF protein and mRNA [66]. With their small size and ability to across BBB, exosomes are important tools to the brain [67]. With the assistance of RVG peptide for neuron targeting, NGF was effectively delivered into ischemic cortex by systemic administration, with a burst release of encapsulated NGF protein and de novo NGF translated from the delivered mRNA, highly stable and efficient for an extended period of time in vivo, and the final NGF concentrations in vesicle suspensions were 2 mg/ml.

NGF incorporated in gelatin nanostructured lipid carriers has the potential to enhance the recovery of SCI [67]. With the assistance of ultrasound, poly nanobubbles expressing NGF can be destroyed, showing effectiveness on nerve regeneration by reducing neuron loss and apoptosis in SCI rats [68]. Under ultrasonic irradiation, 52.7% of NGF was released after 12 h. Taking advantage of the high affinity between choline and choline transporters and utilizing the transport of acetylcholine from the pre- to the post-synaptic neuron through nicotinic acetylcholine receptors, Xu et al*.* encapsulated NGF within nanocapsules through in situ polymerization involving 2-methacryloyloxyethyl phosphorylcholine [10]. In a mouse SCI model, the nanocapsules accumulated and remained in the whole spinal cord with a significant functional recovery in locomotion presented by extensive ankle movements and occasional plantar stepping.

In an optic nerve injury model, Michal et al*.* combined NGF with iron oxide nanoparticles constructing a magnetic field-mediated carrier to target the injured part [69]. The nanoparticles were shown to target and accumulate in the retinal area administered to mice by intravenous injection. By placing a magnet next to the mouse's right eye, the targeted delivery of NGF using iron oxide nanoparticles was achieved.

The nanoparticles by intravenous administration open an alternative avenue for tissue engineering and the treatment of CNS disorders and neurodegenerative diseases. Based on the advantages of the size and surface charge of the nanocarriers, by wrapping NGF inside the nanoparticles, the problem of the BBB can be effectively alleviated, and the treatment of special parts in the brain using NGF. In addition, through extensive research on the BBB, connecting specific brain-targeting ligands with nanocarriers can not only increase the brain uptake of NGF but also minimize drug toxicity and enhance the therapeutic effect of neurological diseases.

### Bioactive scaffolds and nerve guide conduits

Scaffolds are widely used in SCI and PNI treatment. Anti-inflammatory drugs and neurotrophic drugs such as gangliosides are mainly used drugs for SCI and PNI treatment. But in 2002, the American Association of Neurosurgeons published evidence-based guidelines for the treatment of acute SCI stating that its clinical benefits have not been proven. In addition to cell transplantation therapy, molecular therapy and combination therapy, biomaterials and 3D printing technology have also brought new hope. The scaffolds loaded with NGF and antibacterial drugs are of great research value and have the potential for promotion and application. Bridging biomaterial constructs have been designed to promote axon growth after SCI. These bioactive scaffolds can be used as a contact-guided axon growth injury gap and a carrier for delivering neurotrophic factors to modify the microenvironment [70, 71], with The NGF expression peaked at 271.46 pg per 1 mg cell lysate at 8 h, or 200 ng/scaffold. 90% release in 8d and completely release within 40d. Many experts agree that the greatest hope for treating SCI will involve a combination of biomaterial scaffolds, cell transplantation, and molecule delivery. A neurobridge scaffold based on silk fibroin was designed to be implanted, slowly releasing NGF from alginate microspheres to the central lesion site in SCI. Released NGF could increase the preservation of spinal cord tissue and the number of surviving neurons [19]. This optimal multi-disciplinary approach offers a promising treatment for the injured spinal cord. Through electrospray, Song et al. fabricated a sandwich-structured composite membrane with three layers to treat SCI, with 1700 pg/cm2 NGF in scaffold, releasing 380 pg of NGF in 30 days [72]. Polylactic acid film prevented drug diffusion and provided mechanical support, poly(lactic-co-glycolic) acid microspheres loaded and released NGF controllably, and chitosan film seeded bone marrow mesenchymal stem cells. The composite membrane could decrease the administration frequency and improve patient compliance as well as promote PC12 cells to differentiate into neurons.

NGF-loaded heparin/chitosan scaffolds designed by Li et al. may have potential application for peripheral nerve regeneration [5]. They used chitosan, a tissue engineering material that was biocompatible, biodegradable, antibacterial and easy to manufacture. The scaffolds were fabricated via electrostatic interaction and pre-immobilized heparin could enhance the NGF stability in chitosan scaffolds. It has been reported that the binding of growth factor and heparin can significantly enhance its binding ability to cell receptors and improve its stability against proteolytic degradation or degeneration, with 27% NGF release after 24 h, 50% release in the 60th day [73]. As a drug delivery carrier on the scaffold, heparin delivers NGF to target cells and maintained the biological activity of the NGF, successfully filling the critical gap of the scaffold. The scaffolds could improve the attachment, proliferation and morphology development of SCs in vitro. Zhang et al. found that a genipin cross-linked chitosan-sericin scaffold to deliver NGF could support SCs growth with good mechanical properties, including high porosity and swelling ratios, with over 65% release within 4 h; full release after 72 h [74]. In a preclinical chronic nerve compression animal model, it was found that treatment for nerve injury by decreasing neuralgia, improving nerve conduction velocity, accelerating microstructure restoration, and attenuating gastrocnemius muscle dystrophy.

Nerve guide conduits (NGCs) loaded with NGF can provide both topographical guidance and chemotaxis for SCI or PNI treatment, thus enhancing the repair of small nerve gaps. Through the process of electrospinning, an aligned cylindrical matrix was generated to match the shape of the cord and influence the directional growth of axons. Robust cellular infiltration and axon regeneration with directionality occurred in a completely transected rat spinal cord after implantation. Aligned silk fibroin nanofibers loaded with Glial cell line-derived neurotrophic factor and NGF could enhance the length and rate of axonal outgrowth of dorsal root ganglions sensory neurons and spinal cord motor neurons from chicken embryos parallel to the aligned nanofibers in vitro [20]. Zhou et al. harvested and seeded bone marrow-derived stromal cells on NGF-loaded poly(ε-caprolactone) (PCL) nanofibrous NGCs in a rotary cell culture system. Highly aligned PCL fiber conduits coated with NGF through electrospinning could enhance and attract the neurite longitudinal growth of dorsal root ganglion neurons toward their high-concentration gradient side in rat sciatic nerve defects. The bioactivity of NGF was well preserved for as long as 28 days [75].

With the development of tissue engineering technology, artificial nerve grafts based on bioscaffold materials and nerve guide conduits have strong application value, and related research is developing a new generation of neural conduits. Their three-dimensional spatial structure provides a place for nerve cells to acquire nutrients, exchange gases, excrete wastes, grow and metabolize, thereby inducing axons to grow along the artificial nerve grafts. At the same time, by loading NGF, these materials can achieve sustained and controlled release of NGF, so that NGF can have a lasting effect on the injured nerve without side effects. For the above reasons, NGF-loaded artificial nerve grafts are increasingly used in the treatment of peripheral nerve diseases.

### Hydrogel

Hydrogel is a water-insoluble and highly cross-linked system formed by hydrophilic polymers. Due to its high water content and similar mechanical properties to human tissues, hydrogel materials are widely used in biomedical fields such as drug control release, tissue scaffold construction and active cell coating. Injectable hydrogels can potentially mimic natural living tissue and fill shaped defects with minimal invasiveness, having a wide range of applications in neural repair and functional reconstruction. By embedding NGF, an adaptable microporous hydrogel effectively could promote cell migration and induce dramatic bridging effects with the ability to assemble into a complex shape in real-time in vivo. Li et al*.* designed a novel thermosensitive heparin-poloxamer hydrogel co-loaded with basic fibroblast growth factor (bFGF) and NGF to treat PNI with chronic illnesses or diabetes mellitusis [18]. In vitro*,* the vehicle could control the release of growth factors in a steady model and prevent degradation. In vivo, after administering in diabetic rats with sciatic nerve crush injury, it was effective in facilitating SCs proliferation, leading to an increased expression of nerve-associated structural proteins, enhanced axonal regeneration and remyelination, and improved recovery of motor function.

Since injectable hydrogels do not require surgical implantation, the surgical trauma is minimal, and can be filled in tissue defects arbitrarily, attracting more and more researchers' attention in the field of peripheral nerve tissue engineering. At the same time, the injectable hydrogel can be mixed with some biologically active substances such as NGF or cells easily and conveniently before injection, it can be constructed into a hydrogel sustained-release system, so as to achieve the slow and controlled release of the biologically active substances, complete the interaction of biologically active substances with receptors or integrins and realize the treatment of PNI.

NGF delivery strategy is strongly associated with disease area. Nanoparticles are administered mainly by injection and then travel through the bloodstream to reach the lesions. Nanoparticles have certain advantages in brain targeting and crossing the blood–brain barrier. Scaffold, nerve guide conduit and hydrogel are mostly administered through implantation and allow the attachment of therapeutic cells. The advantages include longer dosing intervals and sustained release times, and the corresponding disease area is primarily the spinal cord and sciatic nerve.

## Biosafety of NGF

NGF has good applications in treating central nervous system diseases, PNI, bone regeneration, optic nerve regeneration and skin damage repair. However, as a therapeutic agent, the biological safety of NGF is also a factor that has to be considered. Evidence suggests that NGF may be associated with bronchial asthma. During the onset of asthma and other allergic diseases, mast cells and eosinophils in the airway can synthesize and secrete NGF, thereby increasing the amount of NGF in the body. NGF is a dual regulatory mediator of neuroplasticity and immune regulation and has obvious effects on airway remodeling and airway inflammation in asthma. NGF-induced neurokinin causes neurogenic airway inflammation, alters neuroplasticity, and leads to a variety of pathophysiological changes in asthma. NGF can also induce the proliferation of bronchial smooth muscle cells and aggravate the airway remodeling of asthma. In addition, the airway nervous system could be mediated to regulate the plasticity of neurons, making changes in the anatomical structure and function of the airway. At present, there is no consensus on the lowest dose of NGF to cause asthma. What we can do is monitor asthma disease indicators when treating with NGF.

In addition to causing bronchial asthma, NGF and its receptors have been found to be over-expressed in malignant tumors in the ovaries, breasts, lungs, pancreas, skin, liver, stomach and thyroid. They can autocrine to stimulate the growth and diffusion of cancer cells. In breast cancer, NGF signaling via TrkA can trigger the proliferation and invasion of cancer cells. Additionally, breast cancer stem cells could be activated by NGF-induced epithelial-mesenchymal transition and the increase in the number of symmetric divisions, thereby participating in the self-renewal of cancer stem cells. In ovarian cancer, NGF activates TrkA in granulosa cells, where it acts as an indirect angiogenic factor by increasing the expression of vascular endothelial growth factor, leading to cancer cell proliferation, migration and angiogenesis. Although NGF is associated with tumorigenesis, with advancements in delivery technology, precise drug delivery can also make NGF a potential drug for the treatment of neurological diseases in tumor patients.

## Conclusions and outlook

NGF plays a vital role in neutrophilic and neuroprotective aspects and has shown great potential in neurodegenerative disorders treatment, which makes the growing need for NGF in the clinic. The traditional natural extraction method has a complex extraction and purification process and production efficiency is low, so more and more people turn their attention to recombinant protein production technology to find alternative solutions. As for NGF production, there is a lack of relevant research evidence to show the physiological activity and three-dimensional protein structure of NGF obtained from different recombinant protein expression systems. In terms of cost and industry maturity, *E*. *coli* and yeast expression system has the most application potential and industrial production prospect. However, the *E*. *coli* expression system lacks some pathways and enzymes for post-protein modification in eukaryotic cells. The yeast system has eukaryotic post-translational modifications of the produced protein, although whether it is consistent with the post-translational modification molecules of wild-origin NGF has not been confirmed. The highest protein expression is found in mammalian expression systems, and the advantage lies in potential cell delivery applications rather than protein production and purification. High production cost, long production cycle and difficulty in large-scale production are the urgent problems that mammalian expression systems need to overcome. As for plant expression system, although not fully developed, it has great potential for application in the production of heterologous proteins, as it offers economic and safety advantages compared with conventional production systems. The current problem of the plant system is the low yield and expression efficiency. Some teams have tried to use nanocarrier technology for transfection to solve this problem. At the same time, the use of ultrasound and other physical means to assist gene delivery or target chloroplasts to produce proteins [76] is under study.

Diverse delivery systems such as nanocapsules, hydrogels, scaffolds, membranes and fibers have been designed to suppress NGF degeneration and release sustainably in a controlled manner during a longtime after administered systemically or locally. The intranasal and ocular administration can provide a noninvasive, safe and effective method for the direct delivery of NGF to the brain. As for delivery systems design, enforceable tactics to promote BBB penetration efficiency should be proposed, such as BBB’s hypertonicity improvement and ligand-receptor recognition. Achieving the required therapeutic window is also a difficult task since it requires NGF level in target areas at relevant therapeutic concentration without eliciting undesirable side effects, mainly demonstrated as bronchial asthma and tumors. Therefore, intelligent drug delivery, precise drug delivery, on-demand drug delivery, and improving the bioavailability of NGF are the research directions in the field of NGF delivery in demand. In addition, the development of a new targeted drug delivery system to achieve the sustained and controlled release of NGF, reduce the biodistribution of NGF outside the lesion, and relieve the toxic and side effects of NGF is also a clinical imperative.

In general, NGF-based production and treatment approaches have great potential for the treatment of neurological diseases. In future clinical trials, a better understanding of the onset and progression of neurodegenerative disorders will facilitate timely diagnosis and target selection, which should allow early treatment for certain of these diseases. As well as the complexity and heterogeneity of the disease mechanisms, the safety, efficacy, stability and practicability of NGF delivery systems are also major factors influencing the outcome of application in injury sites of brain, spinal cord, etc. As progress continues in optimizing transgene design, delivery, and vectors, the prospects of NGF gene therapy for neurodegenerative disorders will undoubtedly become even brighter.

## Supplementary Information

**Additional file 1:** **Table S1.** Production strategies and yields of different expression systems for NGF proteins. **Table S2.** Therapeutic strategies for NGF delivery to different lesions.

## Data Availability

Not applicable.

## Abbreviations

NGF: Nerve growth factor
PI3K: Phosphatidylinositol 3-kinase
MAPK: Mitogen-activated protein kinase
PLC-γ: Phospholipase C-γ
SCs: Schwann cells
CNS: Central nervous system
AD: Alzheimer disease
SCI: Spinal cord injury
ICV: Intracerebroventricular
PNI: Peripheral nerve injury
RGCs: Retinal ganglion cells
mNGF: Mouse nerve growth factor
BDNF: Brain-derived neurotrophic factors
NT-3: Neurotrophin-3
NT-4: Neurotrophin-4
hNGF: Human nerve growth factor
rhNGF: Recombinant human nerve growth factor
SF-9: Spodoptera frugiperda
NGC: Nerve guide conduit
PCL: Poly(ε-caprolactone)
bFGF: Basic fibroblast growth factor
MAPK: Mitogen-activated protein kinase
