# Correction to: Challenges and progress of neurodrug: bioactivities, production and delivery strategies of nerve growth factor protein

**DOI:** 10.1186/s13036-025-00543-7

**Published:** 2025-07-28

**Authors:** Nan Zhou, TingWei Gu, Yang Xu, Yuda Liu, LiHua Peng

**Affiliations:** 1 College of Pharmaceutical Sciences, Zhejiang University, 866# Yuhangtang Road, Hangzhou, 310058 China; 2 State Key Laboratory of Quality Research in Chinese Medicine, Macau University of Science and Technology, Macau, China; 3 Jinhua Institute of Zhejiang University, Jinhua, 321299 Zhejiang China

**Correction to**: ***J Biol Eng 17***,*** 75 (2023).***

10.1186/s13036-023-00392-2

The original publication of this article omitted the proper attribution for 2 figures. This correction article is to provide the correct attribution for Figs. 4 and 6 and to provide the reference details of the original sources. The incorrect and correct information is shown in this correction article.

**Incorrect**

**Fig. 4:**
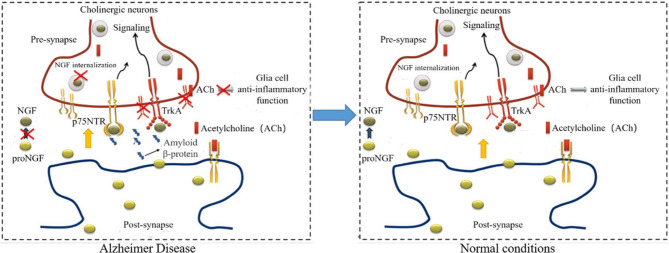
The treatment of NGF for Alzheimer’s disease

**Fig. 6:**
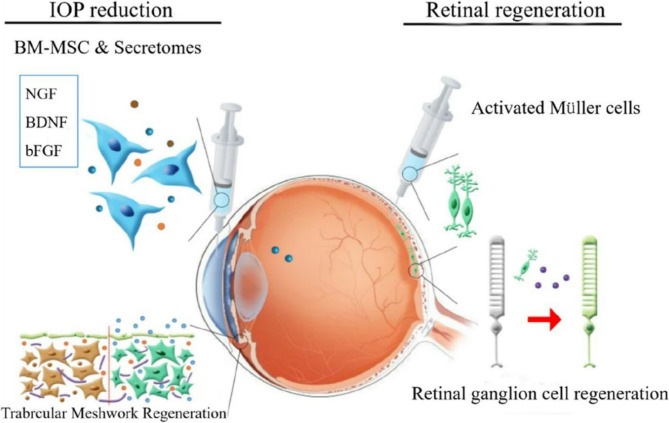
The treatment of NGF for optic nerve regeneration

**Correct**

**Fig. 4:**
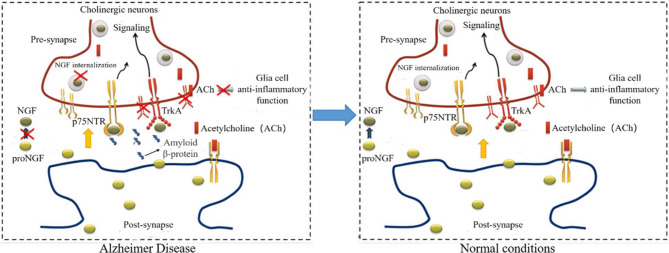
The treatment of NGF for Alzheimer’s disease. **This figure was adapted from Fig. 1 from Mitra et al. (2019)** [77]

**Fig. 6:**
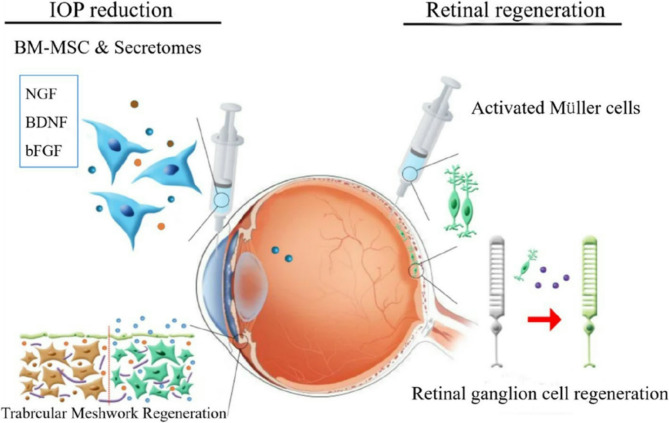
The treatment of NGF for optic nerve regeneration. **This figure was adapted from Fig. 4 from Kwon et al. (2020)** [78]

The original article has been updated. The authors apologize for these oversights and thank the editors, reviewers, and readers for their understanding.
