# Supplementary material for: Challenges and progress of neurodrug: bioactivities, production and delivery strategies of nerve growth factor protein
Source: J Biol Eng. 2023 Dec 4;17:75. doi: 10.1186/s13036-023-00392-2 (PMC10696794; doi:10.1186/s13036-023-00392-2)
Supplement: Supplementary file 1 — Additional file 1: Table S1. Production strategies and yields of different expression systems for NGF proteins. Table S2. Therapeutic strategies for NGF delivery to different lesions. [file 13036_2023_392_MOESM1_ESM.docx]

**Table S1. Production strategies and yields of different expression systems for NGF proteins**

| Expression system | Cell | Transfection vector | Expression | Recovery yield | Purification strategy | Ref. |
| --- | --- | --- | --- | --- | --- | --- |
| *E. coli* | HB101 | \ | 85mg/L | 13% | Guanidine extract; gelfiltration; RPC | 15 |
|  | DE3 | \ | \ | 26mg/L | Isolation and solubilization of IB; guanidine extract; Solid dithiothreitol renature; gelfiltration | 13 |
|  | DE3 | Heat shock | \ | 26% | Affinity chromatography | 17 |
|  | DE3 | Heat shock | \ | 15.30% | Affinity chromatography | 18 |
|  | DE3 | \ | \ | \ | Ultrafiltration; affinity chromatography; gelfiltration | 19 |
| Yeast | S. cerevisiae | Cesium chloride | 5mg/L | \ | \ | 20 |
|  |  | Lithium acetate | 1mg/L | \ | \ | 21 |
| Insect | Sf-9 | Bacmid | 2-10mg/L | 60% | Affinity chromatography | 22 |
|  |  |  |  | 50-60% | IEC; RPC | 23 |
|  |  |  |  | \ | IEC；HIC; RPCE | 24 |
|  |  |  | \ | \ | IEC; gelfiltration | 25 |
| Animal cell | Rat fibroblast | Retroviral vector | 5.4 ng/10^6 cells/24 hr | \ | \ | 26 |
|  | Hela cell | Liposome | 20.3mg/L | \ | \ | 27 |
|  | Mouse NSC | Lentiviral vector | 38ng/mL/mg total protein | \ | \ | 28 |
|  | Rat MSC | Adenoviral vector | 25μg/L | \ | \ | 29 |
|  | Rabbit MSC | Calcium phosphate method | 96.4473pg/10^6 cells | \ | \ | 30 |
|  | Rabbit mammary epithelial cell | Adenoviral vector | 139mg /L | \ | \ | 31 |
|  | Rabbit mammary gland | Adenoviral vector | 173.1mg/L | \ | \ | 31 |
|  | Mice salivary glands | Microinjection | 1.36mg/L | 28mg/40L saliva | Gelfiltration | 32 |
|  | CHO | Liposome | 8.4mg/L | 0.031g/L | IEC; gelfiltration | 33 |
|  |  |  | 17.7μg/L | \ | \ | 34 |
| Plant | Tobacco | Agrobacterium | \ | \ | DEAE anion exchange chromatography; Sp-sepharose cation exchange chromatography; Affinity chromatography | 35 |
|  | Lettuce |  | \ | 0.28mg/g | Sulfate precipitation | 36 |

**Table S2. Therapeutic strategies for NGF delivery to different lesions**

| Nanoparticle | Disease area | Delivery Materials | Adminis-tration | Drug form | Loading and delivering efficiency | Treatment effect | Refs. |
| --- | --- | --- | --- | --- | --- | --- | --- |
|  | \ | Porous silicon film | \ | Protein | Over 90%; Continuous release, with no burst, over 26 d. | Induced neurite outgrowth and profound differentiation of PC12 cells. | 45 |
|  | Brain | Flower-shaped hollow nano-ruthenium | Intravenous injection | Protein | 23%; Penetration of the BBB under the near-infrared irradiation with a release of 67.48% at 42℃ for 48h. | Restored nerve damage, improving learning and memory in AD mice | 46 |
|  | Spinal Cord | Gelatin Nanostructured Lipid Carriers | Intravenous injection | Protein | The final NGF concentrations in vesicle suspensions were 2 mg/ml. | Increased the survival of neurons and improved functional recovery of the acute SCI model rats | 47 |
|  | Spinal Cord | PLGA nanobubbles | Intravenous injection | Protein | 34%; Under ultrasonic irradiation, 52.7% of NGF was released after 12h. | Attenuated histological injury in injured spinal cords, and increased BBB scores in rats with SCI. | 48 |
|  | Spinal Cord | Nanocapsules containing acetylcholine and choline analogues | Intravenous injection | Protein | Vesicles remained in the plasma for up to 10 days, and in the brain and spinal cord tissues for 9 days | Enabled neural regeneration, tissue remodeling, and functional recovery in mice with spinal cord injury. | 4 |
|  | Sciatic nerve; Retina | Iron Oxide Nanoparticles | Sciatic nerve injection; intravenous injection | Protein | Magnetic-field-guided, leading to an accumulation at the magnet site. | Differentiated selected populations of cells in culture. | 49 |
|  | Brain | Exosome | Intravenous injection | Protein; mRNA | 0.87% of total RNA in vesicle was NGF. The NGF expression peaked at 271.46 pg per 1 mg cell lysate at 8 h | Reduced inflammation by reshaping microglia polarization, promoted cell survival, and increased neuroblast | 50 |
| Scaffold | Sciatic nerve | Genipin cross-linked chitosan–sericin 3D scaffold | Implantation | Protein | 200 ng/scaffold. 90% release in 8d and completely release within 40d. | decreased neuralgia, improved nerve conduction velocity, accelerated microstructure restoration, and attenuated gastrocnemius muscles dystrophy of preclinical CNC animal model. | 51 |
|  | \ | Heparin/chitosan scaffolds | \ | Protein | 1700 pg/cm2 NGF in scaffold, releasing 380pg of NGF in 30 days | Improved attachment and proliferation and the morphology development of Schwann cells. | 52 |
|  | Spinal cord injury | PLA/NGF-PLGA/chitosan | Implantation | Stem cell; Protein | 27 % release after 24 h, 50 % release in the 60th day. | Relieved SCI, decreases cavity formation, enhanced neuronal regeneration and tissue repair and locomotor functions. | 53 |
|  | Spinal cord injury | Silk fibroin/alginate neurobridge | Implantation | Protein | Over 65% release within 4 h; full release after 72 h | Enhanced the sparing of spinal cord tissue and increased the number of surviving neurons. | 54 |
| Nerve guide conduit (NGC) | Sciatic nerve | PCL nanofibrous NGC | Implantation | Stem cell; Protein | The bioactivity of NGF was well preserved for as long as 28 days. | Promoted axon elongation and improved functional recovery of rat sciatic nerve. | 55 |
|  | \ | Silk fibroin NGC | \ | Protein | Concomitant release of 6–7 pg/d over 4 weeks. | Determined the direction and extent of axonal outgrowth from DRG sensory neurons and SPC motor neurons. | 56 |
| hydrogels | Sciatic nerve | Anisotropic scaffolds of agarose hydrogels | implantation | Protein | 60 ng release over 18d. | Axons regenerated in animals with anisotropic scaffolds, but not with isotropic scaffold implants. | 57 |
|  | sciatic nerve | Heparin-Poloxamer Thermosensitive Hydrogel | orthotopic injection | Protein | 48% release by day 35. | Facilitated Schwann cell proliferation, expression of nerve associated structural proteins, axonal regeneration and remyelination, and recovery of motor function. | 58 |
